# Diabetic endothelial colony forming cells have the potential for restoration with glycomimetics

**DOI:** 10.1038/s41598-019-38921-z

**Published:** 2019-02-19

**Authors:** Alexander W. W. Langford-Smith, Ahmad Hasan, Ria Weston, Nicola Edwards, Alan M. Jones, Andrew J. M. Boulton, Frank L. Bowling, S. Tawqeer Rashid, Fiona L. Wilkinson, M. Yvonne Alexander

**Affiliations:** 1 Cardiovascular Science, The Centre for Bioscience, Faculty of Science and Engineering, Manchester Metropolitan University, Manchester, UK; 2 Diabetes Research Group, University of Manchester, Manchester, UK; 3 Universiti Kuala Lumpur, Kuala Lumpur, Malaysia; 4 School of Pharmacy, University of Birmingham, Edgbaston, UK; 5 Department of Vascular and Endovascular Surgery, Manchester Royal Infirmary, Manchester University NHS Foundation Trust, Manchester, UK; 6 Manchester Academic Health Science Centre, Manchester, UK

## Abstract

Endothelial colony forming progenitor cell (ECFC) function is compromised in diabetes, leading to poor vascular endothelial repair, which contributes to impaired diabetic foot ulcer healing. We have generated novel glycomimetic drugs with protective effects against endothelial dysfunction. We investigated the effect of glycomimetic C3 on the functional capacity of diabetic ECFCs. ECFCs were isolated from healthy controls and patients with diabetes with neuroischaemic (NI) or neuropathic (NP) foot ulcers. Functionally, diabetic ECFCs demonstrated delayed colony formation (p < 0.02), differential proliferative capacity (p < 0.001) and reduced NO bioavailability (NI ECFCs; p < 0.05). Chemokinetic migration and angiogenesis were also reduced in diabetic ECFCs (p < 0.01 and p < 0.001), and defects in wound closure and tube formation were apparent in NP ECFCs (p < 0.01). Differential patterns in mitochondrial activity were pronounced, with raised activity in NI and depressed activity in NP cells (p < 0.05). The application of glycomimetic improved scratch wound closure *in vitro* in patient ECFCs (p < 0.01), most significantly in NI cells (p < 0.001), where tube formation (p < 0.05) was also improved. We demonstrate restoration of the deficits in NI cells but not NP cells, using a novel glycomimetic agent, which may be advantageous for therapeutic cell transplantation or as a localised treatment for NI but not NP patients.

## Introduction

Diabetic foot ulceration is a chronic complication in diabetes where tissue damage occurs due to neuropathy, ischemia and/or infection^1^ and given its resistance to treatment, provides the impetus for development of novel healing modalities. Chronic wounds are characterized by a persistent inflammatory phase, often complicated with infection, and a failure of defence cell response to damaging micro-environmental stimuli and often results in amputation^2^. One of the notable characteristics of diabetic macroangiopathy (DM), is the prevalence of coexistent coronary disease^3,4^ and vascular calcification^5^, which results in chronic limb ischemia (or CLI) caused by a compromised repair process and ultimately increases risk of mortality^4^. Despite the compromised angiogenic process in diabetes, associated with endothelial dysfunction and microvascular complications^6^, stem or progenitor cell therapy shows promise for repair of ischemic tissue through neovascularisation^7^. A meta-analysis of studies using stem cell therapy, suggests enhanced diabetic foot ulcer healing and outcomes, reducing pain, lowering amputation rate and improving prognosis compared with standard treatment^8,9^. Although there reports demonstrate the impact of endothelial progenitor cells (EPC) in vascular regeneration^10–12^, no studies have evaluated functional distinction between cells isolated from neuroischemic (NI) versus neuropathic (NP) patients.

Both NI and NP patients exhibit neuropathy, which may be caused by a breakdown in homeostatic metabolic and vascular factors, contributing to impaired wound healing through reduced oxygen delivery, nutrients and angiogenic growth factors^13^. The first part of this study aimed to determine whether distinctive differences could be identified between ECFCs isolated from patients with NI vs NP wounds, and establish whether this could contribute to impaired would healing.

Previous work from our group has shown that endothelial colony forming cells (ECFCs) have a defective glycocalyx (reduced 6-*O*-sulfation) in older versus younger people and in turn, compared to cord blood ECFCs^14^. Furthermore, we have also reported restoration of endothelial function in a fatty-acid-induced model of endothelial damage using glycomimetics *in vitro*^15^, supporting other reports, that glycomimetics can improve cell function^16^.

The glycocalyx is composed of glycosaminoglycans (GAGs), which are major components of the extracellular matrix (ECM) and exist as a diverse array of differentially sulfated disaccharide units^17,18^. An important GAG for angiogenesis and wound healing is Heparan Sulfate (HS), and due to its varying sulfation patterning, creates an opportunity to generate smaller, functional HS mimics^19,20^. Glycomimetics can act as regulatory molecules, with targeted therapeutic potential^16^; however, their exploitation has been hindered by the complexity of their synthesis. With recent advances in the construction of synthetic glycans, selective targeting of the ECM has become appealing as a conceivable treatment for a wide range of diseases^21^.

Here, we use ECFCs^12,22^ to identify distinct functional differences between ECFCs isolated from human peripheral blood from NI *vs* NP diabetic patients to (i) determine whether they exhibit similar functional repair defects based on their *in vivo* environment and (ii) establish whether glycomimetic C3 enhances these functional deficits *in vitro*. These findings may allow us to stratify future treatment approaches for this patient group based on their clinical phenotype; NI or NP.

## Results

### Demographics

Patients with type 2 diabetes who were recruited into the study were aged between 59.6 (SD ± 10.4) and 64.7 (SD ± 10.1) years in the NP and NI groups respectively, with the largest proportion being male (79.1%). The mean duration of diabetes was 15.3 (SD ± 10.2) years for NP patients and 14.6 (SD ± 14.6) years for those in the NI group. Routine clinical assessments were performed for participants with diabetes and both NP and NI groups exhibited above normal range levels of HbA1c, LDL and triglycerides, with no significant difference detected between the groups (Additional File Table 1). Furthermore, HbA1c levels appeared to be negatively correlated with the percentage of wound healing (r20.89075, p < 0.0001).

### Distinctive Characteristics of ECFCs from Patients with Diabetes vs Healthy Controls

ECFCs were isolated from NI and NP patients and controls followed by characterisation by positive staining for endothelial cell markers (von Willebrand Factor, CD31 and CD34), lack of a hematopoietic marker (CD45), the ability to incorporate acetylated-LDL and binding of the lectin UEA-1^14^.

**Figure 1:**
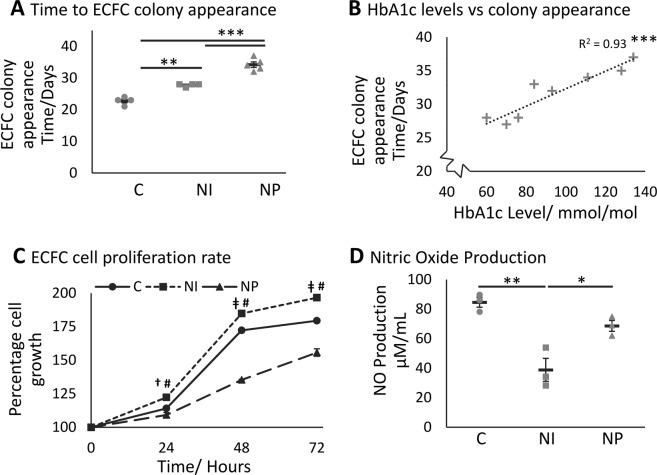
Delayed ECFC colony appearance from patients with diabetes and foot ulcers is related to HbA1C levels and associated with alteration in proliferation rate and nitric oxide production. Time in days from isolation of cells to the appearance of an ECFC colony; a significant delay was identified from controls (C) to patients with neuroischemic (NI) and neuropathic (NP) foot ulcers (**A**) (n = 4, 4, 5 respectively). The time to colony formation significantly correlated with the HbA1C level in patients’ haemoglobin (**B**) (n = 9). The relative proliferation rate of ECFCs after isolation of the first colony was determined using the cleavage of WST-1 every 24 hours for 72 hours (**C**) (n = 3/group). NI (squares and dotted lines) proliferated faster than C (circles and solid line) and grew faster than NP (triangles and dashed line) ECFCs. †indicates p < 0.05 between NI and NP and ǂ indicates p < 0.001 between NI and NP. # indicates p < 0.001 between diabetic groups and control. Nitric oxide production, determined by the Griess assay, was significantly reduced in patients with diabetes (**D**) (n = 3/group). Significance was determined using One-way ANOVA with Tukey *post hoc* analysis and using Pearson’s correlation, *denotes p < 0.05, **p < 0.01 and ***p < 0.001.

Following plating of peripheral blood monocytes (PBMCs), the emergence of the first healthy control ECFC colony was observed between day 21 and 24. This emergence was significantly slower in NI (p = 0.0015; days 27–28) and NP ECFCs (p = 0.00000082; days 32–37), with NP also slower than NI ECFCs (p = 0.00014; Fig. 1A).

A significant positive correlation was apparent between patient blood HbA1c levels and the time of NI and NP colony appearance in culture (R^2^ 0.93; p = 0.00029; Fig. 1B), possibly reflecting poor glucose control in these patients; however, despite the difference in the timing of colony appearance, the conventional “cobblestone” phenotype was observed for all ECFCs groups.

### NP and NI ECFCs Exhibit Differential Proliferative Capacity Compared to Healthy Control ECFCs

The proliferative activity of ECFCs from the 2 diabetic groups and healthy control group was determined (Fig. 1C). NI ECFCs exhibited a significantly higher proliferative rate than control (p = 0.00086; 72 hours), while the NP ECFC proliferation rate was significantly lower than both NI and control ECFCs (p = 0.0000062 and 0.00014 respectively).

### Diabetic Status and Nitric Oxide Bioavailability

Given that endothelial nitric oxide synthase (eNOS) activity is a classic hallmark of endothelial cells, it was hypothesized that ECFCs from patients with diabetes may be defective in their ability to generate NO. Therefore, nitrite and nitrate, stable oxidized products of NO, were measured in conditioned media of ECFCs. A significant reduction in NO production was observed in NI patients vs controls (p = 0.0022) and in NI vs NP patients (p = 0.018; Fig. 1D), with a non-significant reduction in NO bioavailability in NP patients vs control.

### The Chemokinetic Properties of ECFCs are Defective in Patients with Diabetes

**Figure 2:**
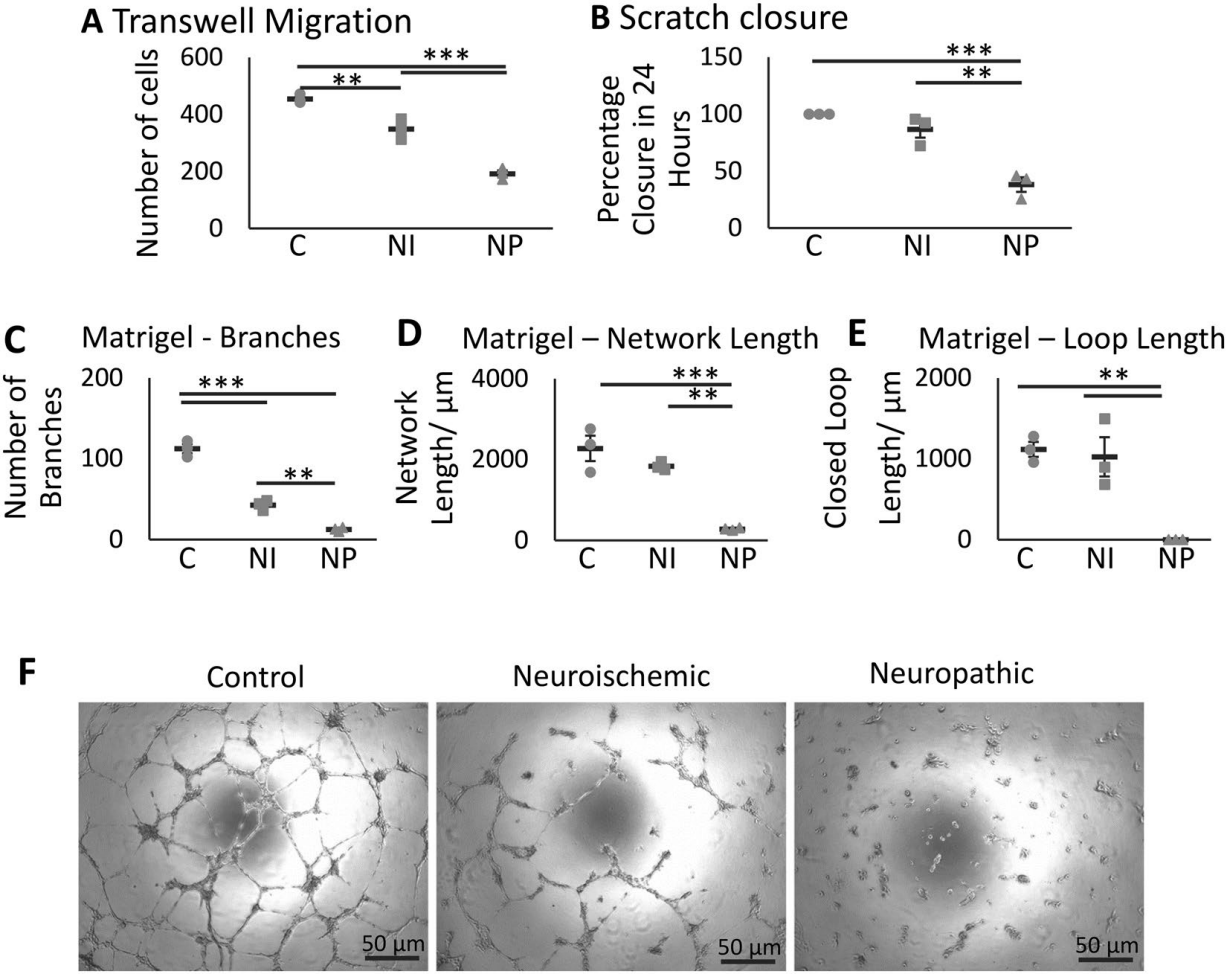
ECFCs from patients with diabetic neuropathic (NP) foot ulcers are more functionally impaired in migration and angiogenesis assays than ECFCs from patients with diabetic neuroischemic (NI) ulcers and control (C). There is a significant decrease in the migration of NI and NP diabetic ECFCs through 8 µm transwell inserts in 6 hours towards 10 ng/ml SDF-1 (**A**) compared to controls (n = 3/group). NP ECFCs are significantly impaired in their migration in the scratch wound assay in 24 hours compared to C and NI (**B**) (n = 3/group). ECFCs were cultured in Matrigel for 8 hours to examine tube and network formation, the number of branches (**C**), length of network (**D**) and closed loop length (**E**) was quantified, identifying functional deficits in the ECFCs from patients with diabetic foot ulcers n = 3/group. Representative images are shown in (**F**), bar = 50 µm. Significance was determined using One-way ANOVA with Tukey *post hoc* analysis, *denotes p < 0.05, **p < 0.01 and ***p < 0.001.

To establish chemokinetic properties of the ECFCs, the Boyden chamber migration assay was employed. The average number of migrated ECFCs for the healthy control, NI and NP groups, without stimulation, were 455 ± 15, 349 ± 36 and 193 ± 19 cells respectively, showing a significantly diminished migration in NI and NP ECFCs compared to controls respectively (p = 0.0050 and p = 0.000034), with NP ECFC migration being significantly reduced vs NI (p = 0.00064; Fig. 2A).

An *in vitro* scratch wound assay was performed to assess the temporal ability of ECFCs from each group to migrate into the wounded site at 0, 6 and 24 hours (Fig. 2B). After six hours, the percentage wound closure was 44.4 ± 1.7% for control ECFCs, with no significant difference to NI (39.1 ± 0.9%). In contrast, NP closure was 27.1 ± 2.4%; significantly less than control and NI ECFCs (p = 0.0011 and 0.0074 respectively; data not shown). Complete wound closure was established after 24 hours for control cells, and although not significant, NI cells showed a small decrease in migration, with only 86.7 ± 7.3% closure compared to controls. However, the NP cells demonstrated a significant decline in migration capacity, with only 38.2 ± 6.4% closure compared to control and NI (p = 0.00057 and 0.0021respectively; Fig. 2B).

### Effect of Diabetic Status on ECFC Angiogenic Capacity

Endothelial tube-forming potential of the ECFCs was assessed by examining network formation (number of branches, length of network, and length of closed loops) in the Matrigel assay (Fig. 2C–F). The number of branches was significantly reduced in NI (42.7 ± 3.5) and NP ECFCs (12.7 ± 1.5) compared to controls (112.3 ± 5.8; p = 0.000043, 0.0000054). The network length for both control (2273 μm ± 315) and NI (1837 μm ± 58) ECFCs was significantly greater (p = 0.00067 and 0.0025 respectively), when compared to the NP group (285 μm ± 17), with no difference observed between control vs NI. However, the loop length from both the control (1115 μm ± 93) and the NI groups (1023 μm ± 244) was significantly larger (p = 0.0047 and 0.0071 respectively) compared to NP cells, where no perimeter length could be quantified. Representative images of the network formation are shown in Fig. 2F.

### Effect of Diabetes on Mitochondrial Activity

**Figure 3:**
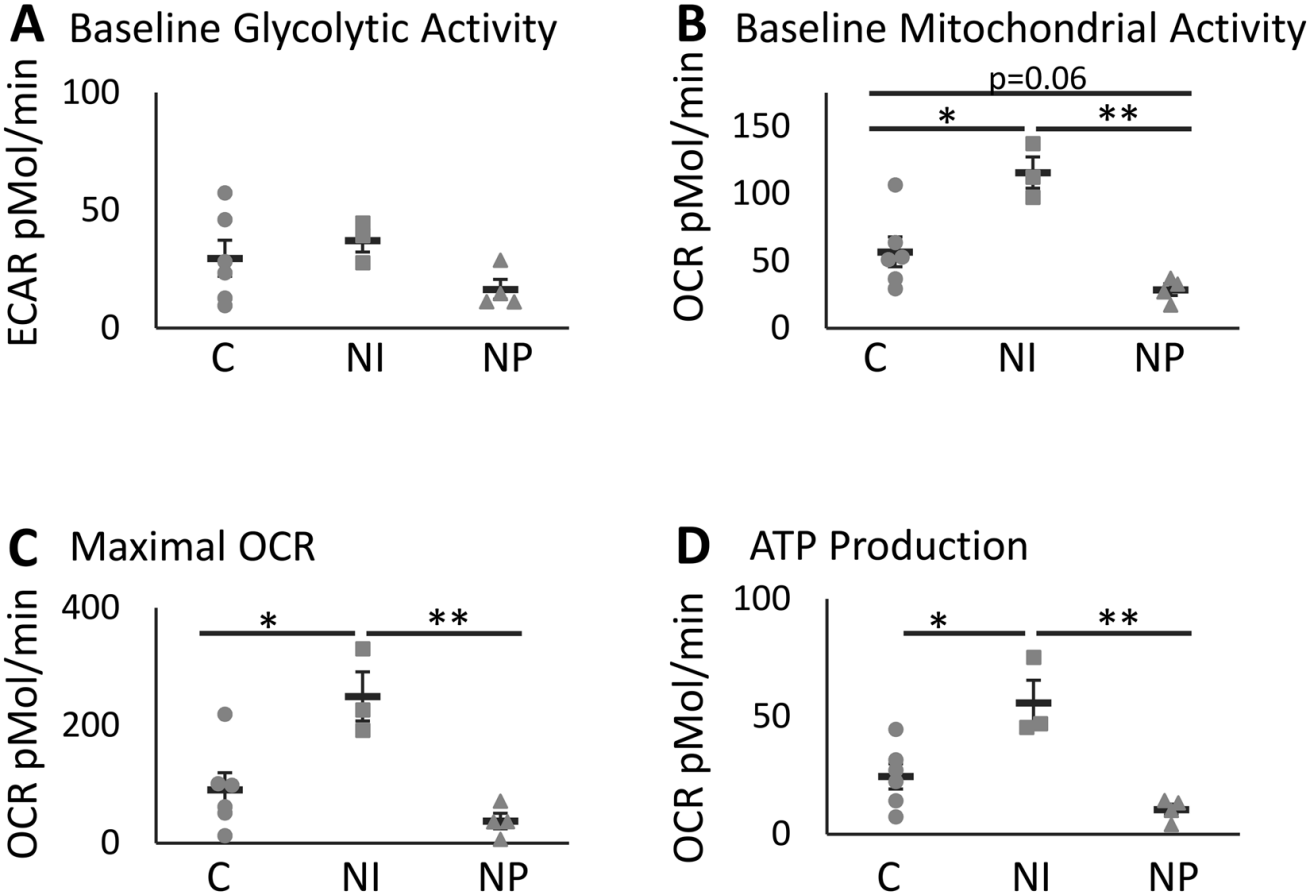
Increased mitochondrial activity and capacity in neuroischemic (NI) ECFCs compared to control (C) and reduced in neuropathic (NP) compared to neuroischemic ECFCs. The baseline extracellular acidification rate (ECAR), a measure of glycolysis, (**A**) and the oxygen consumption rate (OCR), a measure of mitochondrial function, (**B**) was measured in an Agilent Seahorse extracellular flux bioanalyser. After addition of oligomycin, FCCP and rotenone the maximal OCR (**C**) and the amount of ATP produced (**D**) was determined. Significance was determined using One-way ANOVA with Tukey *post hoc* analysis, *denotes p < 0.05, **p < 0.01 and ***p < 0.001. n = 6C, 3 NI and 4 NP.

Given that mitochondria play a key role in cell function, and in order to investigate whether altered mitochondrial activity may account for the differential proliferative capacity and delayed colony appearance of NI and NP ECFCs, we assessed extracellular acidification rate (ECAR), a measure of glycolysis, and oxygen consumption rate (OCR), a measure of mitochondrial activity, using the Seahorse extracellular flux bioanalyser. No significant difference was detected between the baseline glycolytic activity by ECAR in controls, NI and NP ECFCs (Fig. 3A); however, there were differences between all groups in OCR baseline mitochondrial activity. NI ECFCs showed higher activity compared to NP (p = 0.0010) and controls (p = 0.016; Fig. 3B). NP ECFCs were under active (p = 0.59 Tukey *post hoc)*, although when comparisons between control and NI or NP only were considered using the Dunnett *post hoc* test, a significant difference was reached (p = 0.05). In addition, the NI group showed higher activity in maximal consumption of oxygen vs NP (p = 0.0044) and controls (p = 0.024; Fig. 3C). Basal ATP production showed a similar pattern, with significantly impaired ATP production being observed in NP ECFCs (p = 0.0024) compared to NI (Fig. 3D), and increased production in NI compared to control (p = 0.024).

### Analysis of Plasma Angiogenic Factors

**Figure 4:**
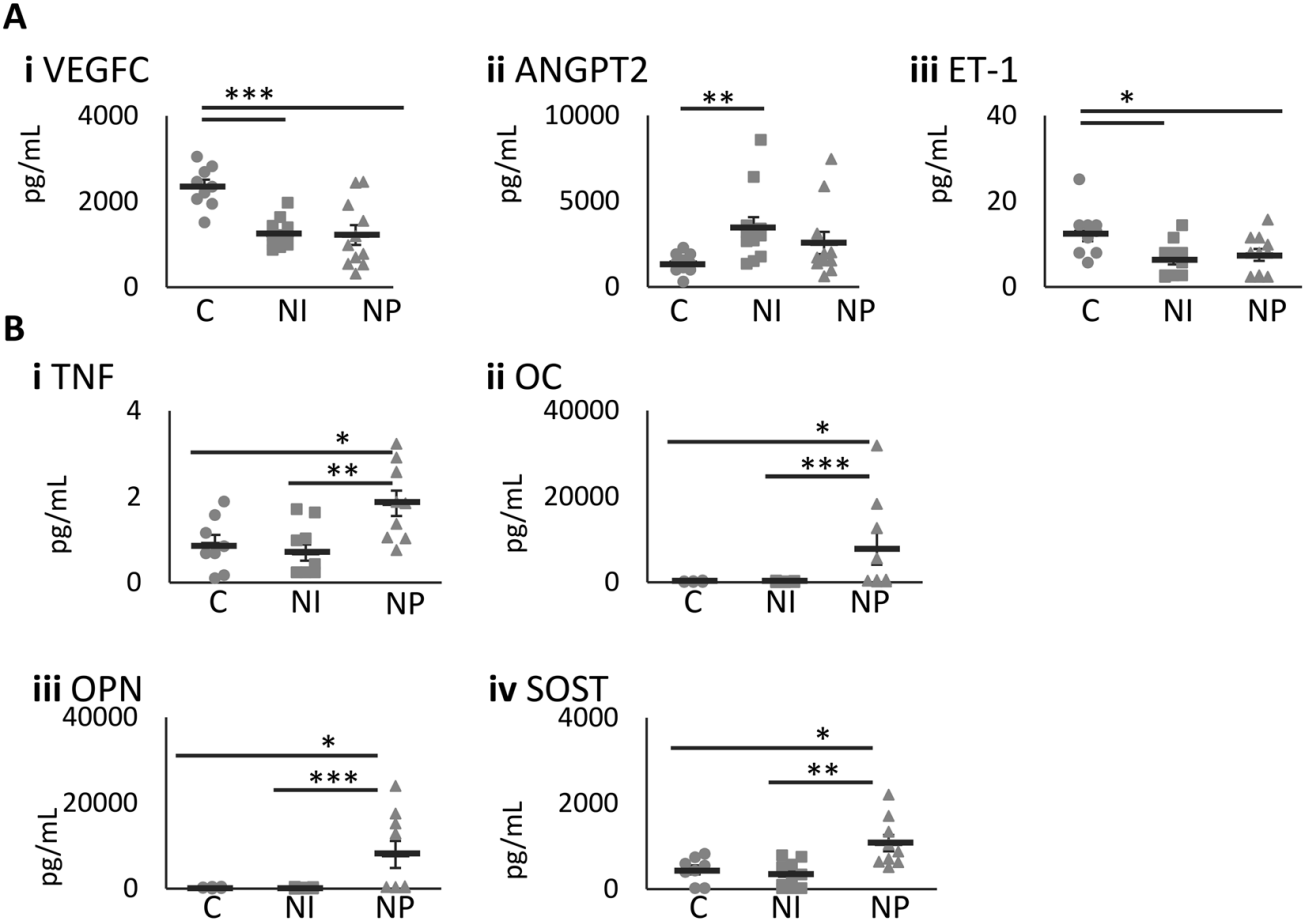
Alterations in plasma inflammatory and angiogenic markers in patients with diabetic foot ulcers compared to control. The levels of angiogenesis markers were measured in n = 10 C, 12 NI and 11 NP plasma samples, vascular endothelial growth factor C (VEGFC) (**Ai**) and Endothelin 1 (ET-1) (**Aii**) were significantly reduced in NI and NP and Angiopoietin-2 (ANGPT2) (**Aiii**) was significantly elevated in NI. The inflammatory cytokine Tumour necrosis factor- α (TNF α) (**Bi**) and Osteocalcin (OC) (**Bii**), Osteoprotegerin (OPN) (**Biii**) and Sclerostin (SOST) (**Biv**) was measured in n = 8 C, 10 NI and 9 NP plasma samples and significantly elevated in NP. Significance was determined using One-way ANOVA with Tukey *post hoc* analysis, *denotes p < 0.05, **p < 0.01 and ***p < 0.001.

Since angiogenic molecules are reduced in Type 2 diabetes^23^, we next investigated whether systemic angiogenic factors are modulated in patient plasma. Of the nine angiogenic markers screened, namely Vascular Endothelial Growth Factor-A (VEGF-A), VEGF-C, VEGF-D, Angiopoietin-2 (ANGPT2), Endoglin, Endothelin-1 (ET-1), Interleukin-8 (IL-8), Hepatocyte Growth Factor and Fibroblast Growth Factor-2, only three were modulated (Fig. 4A). VEGF-C (p = 0.00024 and 0.00027 respectively) and ET-1 (p = 0.013 and 0.048 respectively) were reduced in both NI and NP participants compared to controls; however, no difference was detected between NI and NP patients. ANGPT2 was higher in NI vs controls but no differences were observed between the 2 diabetic groups (p = 0.030).

### Pro-inflammatory and Osteogenic Cytokine Levels in Patients with Diabetes vs Control

Next, it was of interest to determine whether inflammatory or osteogenic-related proteins in the circulation may be linked to the differential ECFC function. We observed a trend towards a reduction in IL-6 in both NP and NI patient plasma compared to healthy controls, but was not significant (data not shown). The level of TNFα is significantly increased in NP, but not NI patients compared to healthy participants (p = 0.028), with a significant difference NI and NP patients (p.0050; Fig. 4Bi). Of the four osteogenic markers screened, OPG showed no significant difference between groups. However, OCN, OPN and SOST were significantly elevated in NP patients compared to NI (p = 0.00066, 0.00065 and 0.0018 respectively) and controls (p = 0.014, 0.013 and 0.013 respectively) (Fig. 4).

### Effect of Glycomimetic Treatment on ECFC Function *In Vitro*

The ultimate goal of the study was to investigate whether the functional defects observed in patient ECFCs could be corrected using our novel glycomimetic, C3, which we reported to rescue endothelial cells from free fatty acid-induced endothelial dysfunction^15^.

**Figure 5:**
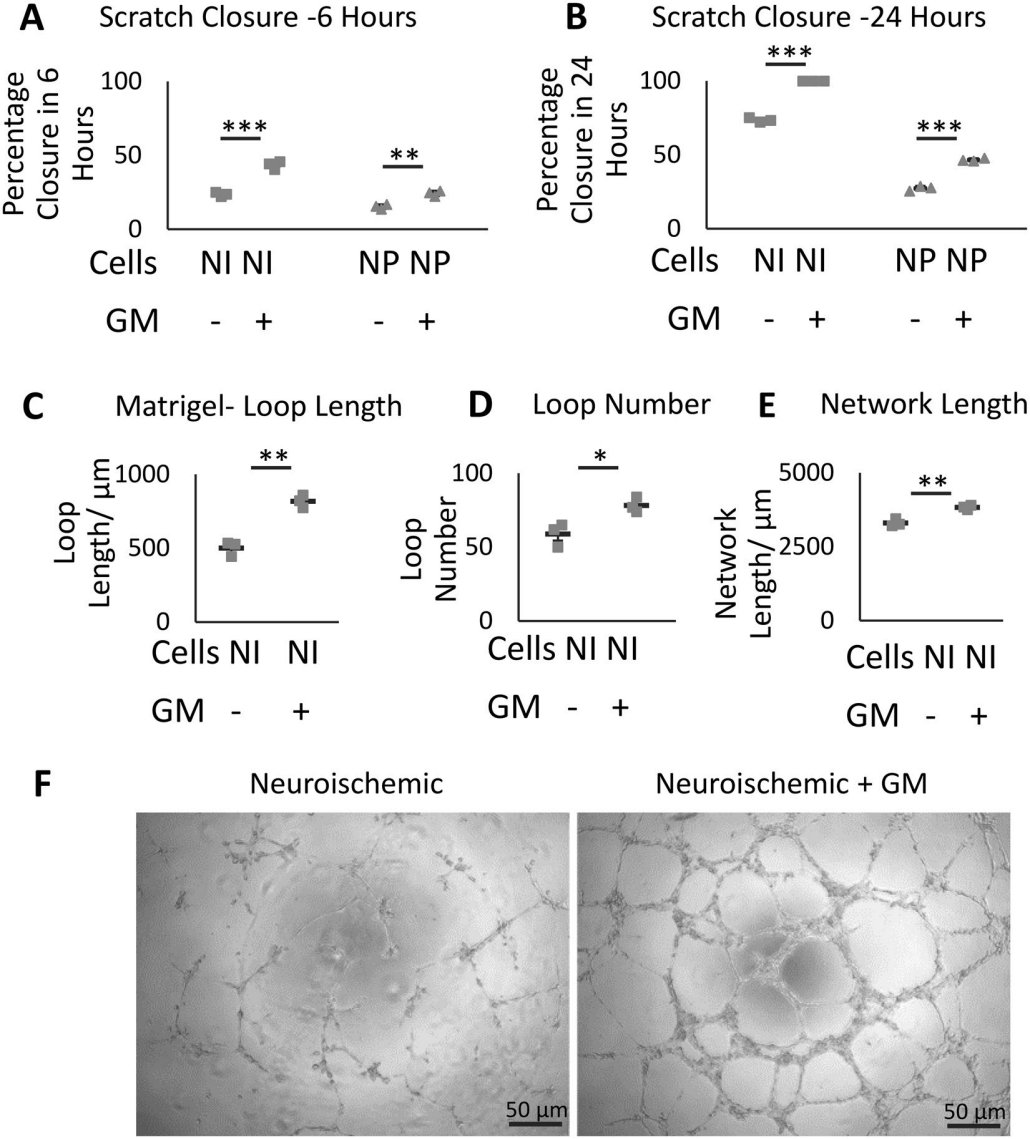
A novel glycomimetic, C3, improved migration and angiogenic capacity of ECFCs from patients with diabetic foot ulcers. There is a significant increase in NI and NP migration in the scratch wound assay at 6 (**A**) and 24 (**B**) hours in the presence of 1 µM glycomimetic C3 (GM) (n = 3/group). NI ECFCs were cultured in Matrigel for 8 hours to examine tube and network formation, the close loop length (**C**), number of loops (**D**) and length of network (**E**) was quantified, identifying a significant improvement in the presence of 1 µM glycomimetic C3 (n = 3/group). Representative images are shown in (**F**), bar = 50 µm. Significance was determined using students T-test, *denotes p < 0.05, **p < 0.01 and ***p < 0.001.

An *in vitro* scratch wound assay was performed, as described above, to establish whether glycomimetic C3 treatment had any effect on the migration. Wound closure was time dependent in both NI and NP groups; after six hours, reached 23.6% and 15.2% for untreated NI and NP ECFCs respectively. However, ECFCs treated with glycomimetic C3, showed improved closure for NI (43.43%) and NP (24.2%) ECFCs (p = 0.00037 and 0.0031 respectively) at 6 hours. After 24 hours, NI attained 73.6% closure, with complete closure following C3 treatment (p = 0.0000071), while NP demonstrated an improvement in closure from 27.3% (untreated) to 45.8% closure following C3 treatment (p = 0.000066; Fig. 5A,B).

### Angiogenic Capacity of ECFCs in the Presence and Absence of the Glycomimetic C3

The matrigel assay was used to determine whether C3 treatment would improve patient ECFCs’ capacity to form an endothelial cell network *in vitro*. NP ECFCs failed to form networks previously and although they were treated with glycomimetic C3, they failed to show any improvement (data not shown) and were therefore omitted from this figure.

A significant improvement in the closed loops perimeter was observed in the glycomimetic C3-treated NI ECFCs (819 µm) compared to untreated NI ECFCs (501 µm; p = 0.0011). Loop number was also significantly improved in glycomimetic C3-treated vs untreated NI ECFCs (78 vs 59; p = 0.024; Fig. 5D). In addition, treated NI ECFCs had longer network length (3835 μm) compared to untreated NI ECFCs (3312 μm; p = 0.0033; Fig. 5E).

Phenotypic observations demonstrated that angiogenic tube formation *in vitro* was enhanced in treated NI ECFCs compared to untreated cells (Fig. 5F), while there appeared to be no effect of the glycomimetic C3 on control cells (data not shown).

## Discussion

Our rationale was to shed light on the cellular properties of ECFCs isolated from two distinct patient groups with a diabetic background; those with NI or NP ulcers. Increasing evidence suggests a role for EPCs in vascular inflammation and endothelial dysfunction, and dysfunctional EPCs can contribute to cardiovascular complications in diabetes^24^, although the mechanism underpinning the impairment remains to be elucidated. With diabetic foot ulceration representing a major cause of patient morbidity worldwide, the limitations in effective treatments provides the drive for innovative therapeutic approaches.

We have previously evaluated both myeloid angiogenic cells^25–27^ and ECFCs^12,14,28^ in the pathophysiology of age and disease. We now demonstrate, that ECFCs from NI and NP patients have distinguishing features and significant defects in their function compared to healthy controls. Furthermore, the NI cells perform significantly better than NP cells in functional assays, as well as in time of isolation and expansion. In contrast, the cells from the patients with NP have very limited capacity for expansion, function or restoration. Therefore, a key finding from this study suggests that NI patients have a greater chance of benefiting from the potential repair capacity of these cells than NP patients.

ECFCs from patients with diabetes exhibited deficits in several parameters; colony appearance, proliferation, migration, chemokinesis, angiogenesis, metabolic function and NO bioavailability. In addition, we found a positive correlation of HbA1c with colony appearance time in NP and NI ECFCs. The influence of high glucose on ECFC function has been thoroughly reviewed^29^, highlighting the links between glucose levels and deficits in number, differentiation, proliferation, adhesion, migration, tube formation, secretome and mobilisation, and an increase in senescence of ECFCs. The data we present here on the inferior performance of the diabetic ECFCs *vs* control cells, is in concordance with the previous reported findings. Interestingly, there is no association between HbA1c, and wound healing in patients with diabetic foot ulcers^30^, suggesting that there are other factors driving this defect in ECFC function. Indeed, we detected no differences in HbA1c levels between NI and NP groups, but this is not surprising given the effective management regime the patients follow for their underlying diabetic condition.

Furthermore, we detected an elevation of TNFα in NP but not NI ECFCs compared to healthy controls, which supports the findings of Chen *et al*., where they demonstrate that TNFα reduces proliferation, migration, adhesion and tube formation of ECFCs *in vitro*^31^. This strengthens our findings of a more severe phenotype in the NP ECFCs *vs* NI ECFCs and the findings of others, who are investigating chronic inflammatory conditions, including systemic lupus erythematosus and rheumatoid arthritis, where ECFCs functional capacity is reduced^32,33^. Furthermore, reports suggest that the close links between redox regulatory pathways and inflammation, may include mitochondrial ROS formation and subsequent eNOS uncoupling^34^. It is also of note, that patients with NI also exhibit NP, therefore additional mechanisms mediated by ischaemia, including stimulation of metabolic pathways, may play a role.

The role of mitochondria in endothelial cell function and vascular disease has been comprehensively reviewed by Tang *et al*.^35^. Our study is the first to analyse the mitochondrial and glycolytic function in diabetic ECFCs and we show an intriguing difference between the NI and NP groups. The NI ECFCs are hyperactive and have an increased total capacity, while the NP ECFCs are underactive. In accordance with NP ECFCs, healthy ECFCs cultured in high levels of glucose^36^ and diabetic PBMCs also have impaired mitochondrial function^37^. The NI ECFCs are not impaired in culture, but are twice as active as the control, indicating they may have adapted to tolerate higher glucose levels. This finding could be explained by a phenomenon known as hyperglycemic memory, where the damaging effects of hyperglycaemia may endure, despite restoration of normal glucose levels^38,39^.

NI ECFCs were found to produce less NO than NP or control, which could be caused by an uncoupling of eNOS, resulting in elevated production of superoxide by the hyperactive mitochondria detected in the NI ECFCs, a reasoning supported by Forstermann *et al*.^40^. The reduced NO generation in the NI ECFCs corresponds to the enhanced proliferation rate of the NI ECFCs observed over control and NP ECFCs. Previous reports suggest a decrease in proliferation in diabetic ECFCs^29,41^; however, healthy cells cultured *in vitro* with high glucose have higher proliferation after a short exposure, yet lower proliferation after longer exposure, this effect was partially mediated by ROS^42^. NO can inhibit and promote endothelial cell proliferation, depending on the vascular bed, the species of cell, or the microenvironment from which the cell originates. It may be that ECFCs from the NI patients acquire an adaptive advantage due to exposure to the hypoxic environment, and thus demonstrate potentiated ECFC proliferation in a NO-independent manner, while the NP cells are more susceptible to hyperoxia-induced growth impairment. Thus, the distinction between the NI and NP ECFC proliferation could be caused by mechanisms other than alteration of NO signaling.

Furthermore, it is also now well established that exposure to a diabetic intrauterine environment increases cardiovascular risk for resulting offspring and there are reports this could be related to impaired function of endothelial progenitor cells (EPCs)^43–45^. It may be that duration of diabetes should have been taken into account when recruiting patients, and in future studies, it would be interesting to know whether the recruited patients were born to mothers with diabetes, as this could be a contributing factor to the functional outcomes of ECFCs, not only in this study but in others also.

Finally, another factor which could have been taken into account was identifying subjects with recurrent foot ulcers, who have been reported to have significantly higher plasma NO compared to subjects with a first time foot ulcer^46^. Since recurrence was not an exclusion criteria in this study, it may be that the NP patients recruited in this study fall into the category of previous ulceration, thus accounting for the higher ECFC NO bioavailability.

Further work is needed to confirm a relationship between the changes in mitochondrial function, NO and ROS in the diabetic setting.

Since changes in the levels of inflammatory mediators are known to influence vascular function and are involved in EPC homing to sites of vascular damage, we investigated the levels of angiogenic, inflammatory and osteogenic proteins and made some key findings, not just within the diabetic environment, but also between the two diabetic groups. ET-1, is a potent vasoconstrictor^47^, but also possesses mitogenic activity^48^ and is involved in the maintenance of cardiovascular homeostasis^49^. We observed a significant decrease of ET-1 in both groups of diabetic patients, potentially contributing to the endothelial injury, poor contractile response and lack of repair observed in the diabetic wound. Whether this could be correlated to the time or effectiveness of wound healing remains to be established. Up-regulation of ET-1 has been shown to correlate with increased circulating EPCs in myocardial infarction and type II diabetes^50,51^, suggesting that the decrease we observe is indicative of a reduction in the number of circulating EPCs and potentially, reduced wound healing capacity. This concept is supported by a study demonstrating a correlation between reduced EPC number and reduced plasma ET-1, with respect to hypertension in children with acute lymphoblastic leukemia, suggesting links between deregulated EPC/ET-1 axis and an impaired post-injury regeneration of the vasculature^52^.

ANGPT2, acts as an inflammatory agent promoting vessel destabilisation^53^ and its elevation has been associated with vascular complications in diabetes^54^. In this study, we show ANGPT2 was significantly elevated in NI patients compared to healthy controls and, although it was higher in NP patient plasma compared to controls, this did not reach significance. It is of interest that ANGPT2 is a key player in the ANGPT2-Tie2 pro-angiogenic pathway and also in the metabolic fitness of a number of cells^55^. Since we found that both angiogenesis and mitochondrial activity were higher in NI ECFCs compared to the NP, these data correlated with the elevated levels of ANGPT2 that were detected in the NI *vs* NP cells.

The pro-angiogeneic factor, VEGFC, was significantly decreased in the diabetic patient group, with no significant difference between the NI or NP groups. Whether this decrease in VEGF-C correlates with reduced wound healing capacity in humans, as has been reported in *db/db* diabetic mice^56^, has yet to be elucidated.

The osteogenic markers, OC, OPN and SOST, were elevated in the NP group of patients compared to both NI and healthy controls. It is interesting that in kidney disease patients, EPCs have been reported to undergo an endothelial-to-procalcific shift, expressing mineralisation biomarkers, including OC and OPN^57^. Flammer *et al*. have also shown that cells expressing both osteogenic and endothelial progenitor cell markers are associated with the presence of elevated HbA1c^58^, which is in accordance with the findings in this study. Since it is known that a positive correlation exists between osteoclast-mediated bone resorption and extra-skeletal ossification^59^, further studies are warranted to discover whether the compromised ECFCs in the NP patients, can be correlated with an increased risk of osteoporotic fractures or the presence of vascular calcification. Furthermore, our data showing elevated OPN levels in the serum of NP patients supports the findings of Wright *et al*., where they demonstrate links between up-regulated OPN and denervated motor and sensory pathways in rats^60^. We also demonstrate a significant elevation in the glycoprotein, SOST, an antagonist of bone formation, which correlates with the elevated levels of OC and OPN observed in this group, and by others^61,62^. However, whether SOST, facilitates communication between the compromised bone microarchitecture and the impaired wound healing found in our respective patient groups remains to be established.

We have previously demonstrated restoration of endothelial cell function, both *in vitro* and *ex vivo*, in a fatty-acid induced model of endothelial damage using glycomimetics and established the effect is executed via upregulation of Akt/eNOS and Nrf2/ARE signalling pathways^15^. Here we show, that glycomimetic C3 improves the migration of both NI and NP ECFCs in a scratch assay; and recovers the angiogenic capacity in NI, but not NP ECFCs. These findings could have implications for the use of these cells prior to transplantation for improved wound healing, or alternatively, the glycomimetic could be delivered systemically to improve endogenous circulating ECFC function. Our data add strength to a previous report by Fraineau *et al*., where the ECFC function was improved *ex vivo* prior to transplantation in a model of hindlimb ischemia, through pharmacologic approaches targeting epigenetic enzymes. The angiogenic repair capacity of ECFCs was shown to be held in a poised state, via gene promoters that are characterized by the presence or absence of active and repressive histone post-translational modifications^24^ controlled by epigenetic enzymes, with competing activities that are co-bound to gene promoters. Since ECFC function is severely compromised in diabetes, the strategy to enhance the angiogenic capacity of these cells and activate improved neovascularisation is an attractive approach to accelerate wound healing in this patient group^10,11^. To our knowledge, this is the first demonstration of a small molecule being used to enhance the function of ECFCs isolated from patients with diabetes and foot ulcers.

**Figure 6:**
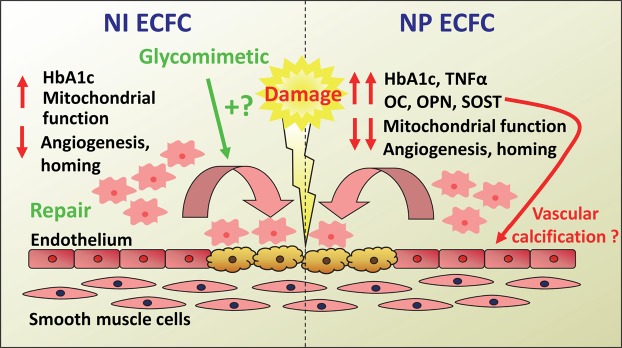
A schematic overview of functional differences in diabetic ECFCs. This figure illustrates the molecular and functional differences between ECFCs isolated from patients with NI (neuroischemic) and NP (neuropathic) foot ulcers, and the potential for restoration of function in NI ECFCs using glycomimetic C3. Glycated haemoglobin (HbA1c); Tumour Necrosis Factor-α (TNFα,); Osteocalcin (OC); Osteoprotegerin (OPN); Sclerostin (SOST).

In summary, our data strengthen previous reports that ECFCs isolated from the diabetic patients are defective in their mobilization, migration, proliferation and tube formation *in vitro* compared to healthy controls, but we also identify distinct deficits in both the NI and particularly in the NP cells summarised schematically in Fig. 6. The defective functional NP repair capacity suggests they are inappropriate for transplantation or drug-induced improvements. Although the NI ECFCs are less impaired then the NP cells *in vitro*, they may have a reduced response to growth factors and *in vivo* are unable to migrate to the sites of damage due to the impaired circulation observed in NI patients. Therefore, interventions to improve blood flow would likely be beneficial as the NI ECFCs have preserved, albeit reduced, healing potential. There were also distinct differences in cytokine profiles in NI and NP patients and healthy controls, which may impact on wound healing capacity. Finally, the migration and angiogenic effects of ECFCs showed significant improvement in NI cells but not NP cells in the presence of glycomimetic C3, suggesting a greater defect in NP ECFCs which was incapable of restoration.

Other studies are already being carried out elsewhere, proving cell therapy to be safe and free from toxic effects, providing a sound foundation for the development of preclinical and clinical trials for the management of ischemic disease^63^. Therefore, identifying the root cause of the impaired function of ECFCs is a key area for further study. These cells show promise in being capable of functional enhancement by our glycomimetic drug; however, future work will focus on animal studies to validate these *in vitro* data and to identify the mechanism of action and the receptor interaction involved. An understanding of the cytokines released at the wound site and the distinction between healing and non-healing ulcers may also help in preventing the progression of ulceration. Although a few challenges related to ECFC-based therapies still exist, further studies in this field will no doubt add strength to the potential use of endothelial progenitor cells for wide-ranging translational significance in regenerative medicine.

## Methods

### Study participants

The study was approved by The Institutional Review Board at the University of Manchester, and the Central Bristol Research Ethical Committee (17/NI/0238); informed consent was obtained from all participants (156412) and the study was performed in accordance with the relevant guidelines and regulations. Patients with type 2 diabetes and foot ulcers were characterised as NP (disability score =≥ 6 [severe] and quantitative sensory testing [vibration perception threshold]^64^) or NI (Ankle Brachial Pressure Index =< 0.7, or Toe Pressure =< 50 mmHg or Transcutaneous Oxygen Pressure =< 30 mmHg or Ankle Pressure =≤ 70 mmHg; Additional File Table 1). Patients were excluded based on lower limb amputation; renal impairment with eGFR <30 ml/min; kidney transplant; rheumatoid arthritis, malignancy, pregnancy and lack of consent.

### Isolation of Endothelial Colony Forming Cells

ECFCs were isolated from peripheral blood from controls and patients, as previously described^14^ and the successful yield of ECFC colonies was from individuals was 80%. ECFCs were cultured in Endothelial Basal Medium MV2 (PromoCell) and used between passages 2–5 (n = 3–12) with and without the addition of 1 µM glycomimetic C3^15^.

### Relative Cell Proliferation Assay

Proliferation assays utilised the “Quick Cell Proliferation Assay Kit” (Abcam). In brief, 1 × 10^4^ ECFCs were cultured per well in a 96 well plate, and the percentage cell growth was calculated during a 72 hr time-course (from 0, 24, 48 and 72 hours) by measuring the absorbance at 650 nm, which reflected WST-1 cleavage (Quick Cell Proliferation Assay Kit, Abcam) and presented as % cell growth/hour.

### Nitric Oxide Griess Assay

NO production was determined by measuring nitrite levels in the media using the Griess method^65^. 5 × 10^4^ ECFCs were cultured in each well of a 96 well plate and the media analysed after 24 hours. 150 µl cell culture supernatant, 130 µl deionised water and 20 µl Griess reagent (Molecular Probes) were incubated at 37 °C for 15 minutes before measurement of absorbance at 540 nm. Nitric oxide production was calculated compared to a known concentration standard curve.

### Transwell Migration Assay

Migration of 5 × 10^5^ ECFCs towards 10 ng/ml SDF-1 through 8 µm collagen-coated transwell inserts was assessed over 6 hours^14^. The number of migrated cells were counted after 0.2% trypan blue staining in 5 fields of view/insert.

### Scratch Cell Migration Assay

A scratch was made in a confluent monolayer, the cells washed twice and the migration of cells into the denuded area was assessed after 0, 6 and 24 hours by light microscopy^14^. The percentage “wound” closure was calculated vs 0 hours.

### Matrigel *in vitro* Angiogenic Tube Formation Assay

1 × 10^4^ ECFCs were cultured in the each of the wells of a 96 well plate in Matrigel (Corning), incubated for 6 hours at 37 °C, 5% CO^2^ and imaged using a light microscope. The number of branches and loops were counted and network and closed loop lengths were measured using ImageJ.

### Metabolic assessment

Glycolysis and mitochondrial respiration rate was determined from the extracellular acidification rate and the oxygen consumption rate using a Seahorse Extracellular Flux Bioanalyser (Agilent). Metabolic assessment was carried out as described in the Mitochondrial Stress Test Kit (Agilent). In brief, 8 × 10^3^ cells were cultured in XFp cell culture miniplates for 16 hours, before washing and equilibrating at 37 °C and atmospheric CO_2_ in XF Base assay medium containing 1 mM pyruvate, 2 mM glutamate and 10 mM glucose. The baseline metabolic activity, ATP production, proton leak, maximal respiration and spare capacity was then measured and calculated in the presence of 1 µM Oligomycin, FCCP 1 µM and 0.5 µM Rotenone.

### Secretome Analysis

The Human Angiogenesis/Growth Factor Magnetic Bead Panel 1 and Bone Panel Milliplex Assays (Millipore) were used to analyse participant plasma according to manufacturer’s instructions.

### Statistics

Significant differences (p ≤ 0.05) between groups was determined using students T-test or One-way ANOVA with Tukey post hoc analysis and correlations using the Pearson’s test.

### Ethics approval and consent to participate

The study was approved by The Institutional Review Board at the University of Manchester, and the Central Bristol Research Ethical Committee (17/NI/0238); informed consent was obtained from all participants (#156412).

## Supplementary information

Supplementary Table 1

## Data Availability

The datasets used and/or analysed during the current study are available from the corresponding author on reasonable request.
