# Supplementary material for: Diabetic endothelial colony forming cells have the potential for restoration with glycomimetics
Source: Sci Rep. 2019 Feb 19;9:2309. doi: 10.1038/s41598-019-38921-z (PMC6381138; doi:10.1038/s41598-019-38921-z)
Supplement: Supplementary file 1 — Supplementary Table 1 [file 41598_2019_38921_MOESM1_ESM.pdf]

Diabetic Endothelial colony forming cells have the potential for restoration with glycomimetics

Alexander W.W. Langford-Smith[1]*, Ahmad Hasan[2,3]*, Ria Weston[1], Nicola Edwards [1], Alan M. Jones[4], Andrew J.M. Boulton[2,6], Frank L. Bowling[2,5,6], S. Tawqeer Rashid[2,5,6], Fiona L. Wilkinson[1,] and M. Yvonne Alexander[1,6].

[1] Cardiovascular Science, The Centre for Bioscience, Faculty of Science and Engineering, Manchester Metropolitan University, UK

[2] Diabetes Research Group, University of Manchester, UK

[3] Universiti Kuala Lumpur, Malaysia

[4] School of Pharmacy, University of Birmingham, Edgbaston, UK

[5] Department of Vascular and Endovascular Surgery, Manchester Royal Infirmary, Manchester University NHS Foundation Trust, UK

[6] Manchester Academic Health Science Centre, UK

[*] These authors contributed equally to this manuscript

Supplemental Table 1: Patient demographics and clinical biochemistry.

Variable	NP (Mean±SD) n=11	NI (Mean±SD) n=13	p Value
**HbA1C**	86.2 ±34.7	71.6 ±12.03	ns
**HDL**	1.18 ±0.37	1.22 ±0.29	ns
**LDL**	1.87 ±0.98	1.96 ±0.82	ns
**Total cholesterol**	3.95 ±1.01	4.26 ±0.98	ns
**Triglycerides**	1.92 ±0.33	2.5 ±0.53	ns
**ABPI**	1.12 ±0.06	0.56 ±0.05	p<0.0003
**NDS**	8.72±1.27	4.58 ±2.31	p<0.0005
**Toe pressure**	0.75 ±0.20	0.48 ±0.19	ns
**TcPO2**	74.2±8.87	43.2 ±9.66	p<0.05

Data were analysed by Mann-Whitney U statistical analysis. HbA1c (glycated hemoglobin), HDL (High Density Lipoprotein), LDL (Low Density Lipoprotein), ABPI (Ankle Brachial Pressure Index) NDS (Neuropathy Disability Score), TP (Toe Pressure), TcPO2 (Transcutaneous Oxygen Pressure), SD (Standard Deviation). Normal Range for HbA1C is <42.0mmolmol. ns; not significant.
